# Liquid Chromatography‒Tandem Mass Spectrometry Analysis of Primary Metabolites and Phenolic Acids Across Five Citrus Species

**DOI:** 10.3390/cimb47040223

**Published:** 2025-03-26

**Authors:** Yujiao Peng, Xueyu Cui, Manman Sun, Xiaojuan Huang, Ke Tang, Baoqing Hu, Hongze Liao

**Affiliations:** 1 Key Laboratory of Beibu Gulf Environment Change and Resources Utilization of Ministry of Education, Nanning Normal University, Nanning 530001, China; yaoyuan200452@163.com (Y.P.); 20180702@nnnu.edu.cn (X.C.); hbq1230@nnnu.edu.cn (B.H.); 2 Guangxi Key Laboratory of Polysaccharide Materials and Modification, School of Marine Sciences and Biotechnology, Guangxi Minzu University, 158 West Daxue Road, Nanning 530008, China; 15237375902@163.com (M.S.); xiaojuanhh@163.com (X.H.); 3 Heilongjiang Academy of Agricultural Sciences Rural Revitalization Science and Technology Research Institute, 800 Innovation Road, Harbin 150086, China; 19841102@163.com

**Keywords:** citrus pulp, citrus varieties, lipids, primary metabolites, phenolic acids

## Abstract

Citrus is a globally consumed fruit with great popularity, yet systematic analyses of primary metabolites across major varieties remain scarce, with phenolic acids as an auxiliary focus due to their flavor relevance. In this study, the primary metabolites and phenolic acids of five citrus varieties were analyzed via liquid chromatography‒tandem mass spectrometry (LC–MS/MS). The analysis revealed that five different citrus varieties contained 342 primary metabolites and 77 phenolic acids. The PCA clearly distinguished the metabolites of various citrus varieties. Compared with the pomelo group, the lemon group presented the most differentially abundant metabolites, whereas the kumquat and navel orange groups presented the fewest. An examination of metabolic pathways with notable disparities revealed that phenylpropanoid biosynthesis and the biosynthesis of amino acids significantly varied between varieties. This study elucidates primary metabolic networks underlying citrus flavor diversification, with phenolic acids providing secondary modulation insights. Moreover, this study provides a theoretical foundation for enhancing the flavor of citrus fruits.

## 1. Introduction

Citrus, which belongs to the Rutaceae family, is extensively grown and has great economic importance worldwide. The fruits are prized not only for their distinct flavors but also for their rich content of primary metabolites (e.g., sugars, amino acids, lipids, vitamins) [1,2]. These compounds not only supply essential energy and nutrients for human activities but also possess the capacity to treat different ailments, including diabetes [3], osteoporosis [4], and colon cancer [5]. Their cultivation is economically significant in several nations, providing support to a vast number of agricultural businesses and trade networks [6,7].

Primary metabolites are fundamental substances synthesized during plant growth, directly governing fruit edibility, flavor, and nutritional quality [8]. Sugars, acids, lipids, and nucleotides are vital metabolites in plants that play a critical role in supplying necessary compounds and energy throughout the plant’s life cycle, influencing both maturity and senescence [9]. The composition of these metabolites is often influenced by factors such as the production environment, harvest season, and citrus variety [10]. Despite the existence of several citrus fruits, systematic comparisons of primary metabolite composition across major varieties remain limited, particularly in pulp tissues. Therefore, elucidating the primary metabolic networks underlying flavor differentiation in key citrus varieties is critical for guiding targeted breeding and processing strategies.

Advancements in technology have resulted in an increasing amount of research that uses metabolites to uncover discrepancies in the quality of food and drugs. Studies using liquid chromatography‒mass spectrometry (LC‒MS) have shown that fluctuations in polyphenol concentrations significantly affect the nutritional value of both fresh apples and dried apples [11]. LC‒MS research on several loquat types has shown that changes in loquat flavor may be attributed to variations in the content and quantity of carbohydrates, organic acids, amino acids, and phenolic chemicals [12]. Wang et al. analyzed the flavonoid compound diversity of 62 citrus varieties, including sweet oranges, lemons, pummelos, and grapefruits, using LC‒MS/MS analysis [13]. In a more recent study, Peng et al. detected 623 metabolites in “Shatian Yu” (*Citrus maxima*) from different regions using UPLC–MS/MS [14]. Previous research has shown that analyzing differences in metabolites is a useful method for identifying variations in fruit quality [15,16,17]. While recent large-scale studies, such as Liang et al., have provided valuable genomic and metabolomic insights into bioactive compound variations in citrus peels across 299 citrus accessions [18], comprehensive investigations comparing the targeted metabolites in the citrus pulp of specific commercially dominant citrus varieties remain limited. Specifically, the functional and flavor-related metabolic differences among key cultivated species have not been systematically characterized using advanced LC‒MS/MS platforms.

Sweet orange (*Citrus sinensis*), pomelo (*C. maxima* var. *shatinyu*), lemon (*C. limon* (L.) *Burm. F.*), kumquat (*C. japonica* (Lour.) Swingle), and orah mandarin (*C. tangerina*) (Figure S1) are the main cultivated and economically valuable species in China. The five citrus varieties display unique sensory attributes. Specifically, sweet orange is recognized for its harmonious sweetness and gentle acidity, accompanied by a fragrant, fruity scent and succulent, tender segments [19]. Pomelo possesses a delicate sweetness, gentle tartness, and a fragrant yet subtle floral aroma, featuring thick, juicy segments that are less fibrous than those of oranges [20]. Lemon possesses high acidity and a tangy profile, characterized by a sharp, zesty taste and a pronounced citrus aroma [21]. Kumquat presents a distinctive amalgamation of sweet rind and tangy pulp, characterized by a crunchy texture and a potent citrus aroma, setting it apart from larger citrus varieties [22]. The orah mandarin is distinguished by its remarkable sweetness, minimal acidity, and delightful citrus fragrance, featuring soft, seedless segments that are easily separable and consumable [23]. This research examines the metabolites of five citrus varieties, identifying their key distinctions and offering a theoretical framework for comprehending the variances in nutritional value and potential avenues for enhancing diverse citrus species.

## 2. Materials and Methods

### 2.1. Plant Materials

Five citrus varieties—sweet orange, pomelo, lemon, kumquat, and orah mandarin—were cultivated on an experimental farm located in Rongzhou, Guangxi, China (22°51′35″ N 110°33′07″ E). All the citrus fruits used in this study were grown from seedlings and were not grafted onto rootstocks. Our work specifically targets five major commercial citrus varieties grown under standardized agricultural conditions. The plants were cultivated under uniform standardized management practices, with fertilization consisting of fermented soybean-based organic manure. The fruits were harvested at their edible maturity stage, which was consistent across all the samples. Three biological replicates were collected per sample. The pulp and juice from the equatorial area of the fruits from the equatorial area were collected as a single biological sample and then placed in liquid nitrogen. The samples were then transferred to a freezer set at −80 °C.

### 2.2. Extraction Process for Metabolite Analysis

The fruit pulp samples were pulverized into a fine powder via liquid nitrogen and then completely blended. Each powdered sample, 100 mg in weight, was mixed with 1.0 mL of extraction solution, which consisted of 70% aqueous methanol. The samples were then extracted overnight at a temperature of 4 °C on a revolving wheel. To ensure full extraction, the samples were vortexed three times throughout this process. Following extraction, the mixtures were subjected to centrifugation with a force of 10,000 times the acceleration due to gravity for 10 min. The resulting supernatants were then separated and passed through a filter (SCAA-104, with a particle size of 0.22 μm; ANPEL, Shanghai, China) before analysis via LC‒MS/MS.

### 2.3. LC–MS/MS Conditions

The fruit extracts were qualitatively analyzed using the high-resolution mass spectrometer AB sciex TripleTOF6600 (AB SCIEX, Singapore City, Singapore), and then they were quantitively analyzed via a liquid chromatography (LC) system (SHIMADZU Nexera X2, Shimadzu, Tokyo, Japan) coupled to a mass spectrometer (SCIEX 4500, QTRAP, Sciex, Redwood City, CA, USA). The column was an SB-C18 chromatographic column (2.1 mm × 100 mm, 1.8 µm). Mobile phase A consisted of ultrapure water with 0.1% formic acid, and mobile phase B consisted of acetonitrile with 0.1% formic acid. The A/B (*v*/*v*) gradient program was as follows: 95:5 (*v*/*v*) at 0 min, 5:95 (*v*/*v*) at 9.0 min, 5:95 (*v*/*v*) at 10.0 min, 95:5 (*v*/*v*) between 11.0 and 4.0 min. The flow rate was 0.35 mL/min, and the column temperature was 40 °C. The injection volume was 4 μL.

The electrospray ionization source temperature was 550 °C; the ion spray voltage for the positive mode was 5500 V, while the value for the negative mode was −4500 V; the ion source gases I and II and the curtain gas were set to 50, 60, and 25 psi, respectively; and the collision-induced dissociation parameters were set to high. Triple quadrupole scanning was performed in multiple reaction monitoring mode, with the collision gas (nitrogen) set to medium. In the triple quadrupole, each ion pair was scanned and detected on the basis of the optimized declustering potential and collision energy. The quality control (QC) samples, which were prepared by mixing all the samples, were analyzed once every ten samples. During the sample analysis, a blank sample was inserted before the QC samples to monitor potential sample carryover or contamination that could affect the results.

### 2.4. Analysis of Metabolites

Metabolites were identified and semi-quantified using the MetWare database (MWDB, Maiwei Biotechnology Co., Ltd., Shenzhen, China) and a triple quadrupole mass spectrometry platform. For qualitative identification, the metabolites that were first excluded were isotopic signals, redundant adducts (e.g., K^+^, Na^+^, NH_4_^+^), and fragment ions from larger molecules to minimize false positives. Then, the metabolites were matched to the MWDB by comparing experimental data—including the exact mass, MS/MS fragmentation patterns, isotopic distribution, and retention time (RT)—with reference entries in the database using a proprietary intelligent MS/MS spectral matching algorithm. Following the acquisition of metabolite spectrum analysis data from several samples, the peak regions of all the mass spectrum peaks corresponding to substances were integrated. The peak intensity cut-off for detected metabolites was 1000. Additionally, the integration of mass spectrum peaks for the same metabolite across multiple samples was corrected [24]. Software, namely MultiaQuant (SCIEX), was used to process the mass spectrometry data. Multivariate statistical analyses, such as a principal component analysis (PCA) and a cluster analysis, were employed to analyze the metabolites. The stability and reliability of the predictive model were assessed via an orthogonal partial least squares discriminant analysis (OPLS-DA) [25]. Differentially abundant metabolites were screened on the basis of variable importance in projection (VIP) values, univariate statistical *p*-values, and fold changes (FCs). The screening threshold for differential metabolites was FC ≥ 2 or FC ≤ 0.5, VIP > 1, and an FDR-adjusted *p*-value of < 0.05. The metabolite abundance data were normalized using Z-score standardization (auto-scaling) to account for technical variability and enable cross-sample comparisons. For each metabolite, raw values were centered by subtracting the mean abundance across all the samples and scaled by dividing by the standard deviation, resulting in a normalized distribution with a mean of 0 and a standard deviation of 1. The metabolites were annotated using the KEGG compound database (http://www.kegg.jp/kegg/compound/, accessed on 13 March 2025) with a focus on plant-derived metabolites. A pathway enrichment analysis was performed by mapping the annotated metabolites to the KEGG pathway database (http://www.kegg.jp/kegg/pathway.html, accessed on 13 March 2025) using custom R scripts (R v3.5.1; ggplot2 v3.3.0 for visualization) [26].

## 3. Results

### 3.1. The Analysis of the Metabolites Among Five Citrus Varieties by LC–MS/MS

**Figure 1:**
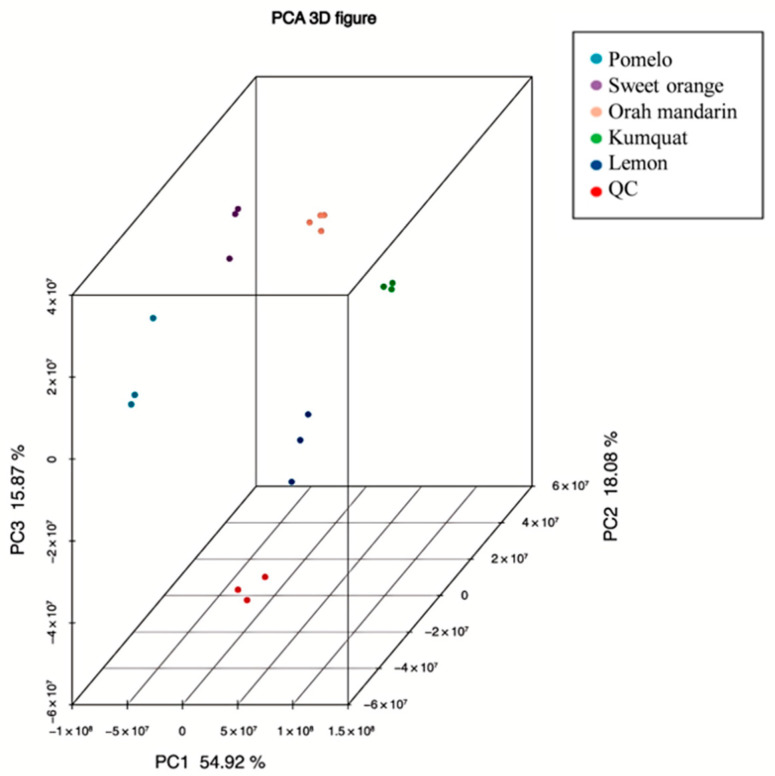
The PCA score plot of the metabolites in the five citrus varieties.

In five citrus fruits, a total of 419 metabolites were detected (Table S1), which were classified into 5 primary categories and 11 secondary categories (Table 1). The three biological replicates of each variety formed distinct clusters (Figure S2). The total ion chromatograms (TIC) for the LC‒MS of the mixed samples from the negative and positive ion modes are shown in Figure S3. The pomelo and sweet orange samples presented the greatest number of identified metabolites, with a total of 418, whereas the kumquat samples presented the lowest number, with 387. Among the 11 secondary categories, there were no differences in the metabolite numbers of phosphatidylcholine and lysophosphatidyl choline across the studied citrus samples. A PCA was conducted on different citrus samples (Figure 1). The contribution rates of PC1, PC2, and PC3 were 54.92%, 18.08%, and 15.87%, respectively, accounting for 88.87% of the total variance. The relatively concentrated distribution of the samples within each group indicated good experimental repeatability. The clear divergence patterns observed across the sample groups indicated variations in metabolites among the various citrus varieties.

### 3.2. Differential Metabolites in Different Citrus Varieties

To elucidate the primary metabolite composition patterns among citrus varieties, we performed a metabolic profiling comparison across five different citrus varieties (Table 2). The group with the fewest differential metabolites was kumquat vs. sweet orange, with a total of 39 significantly different metabolites identified. The group with the most differential metabolites was the lemon vs. pomelo group, with a total of 67 significantly different metabolites identified.

**Figure 2:**
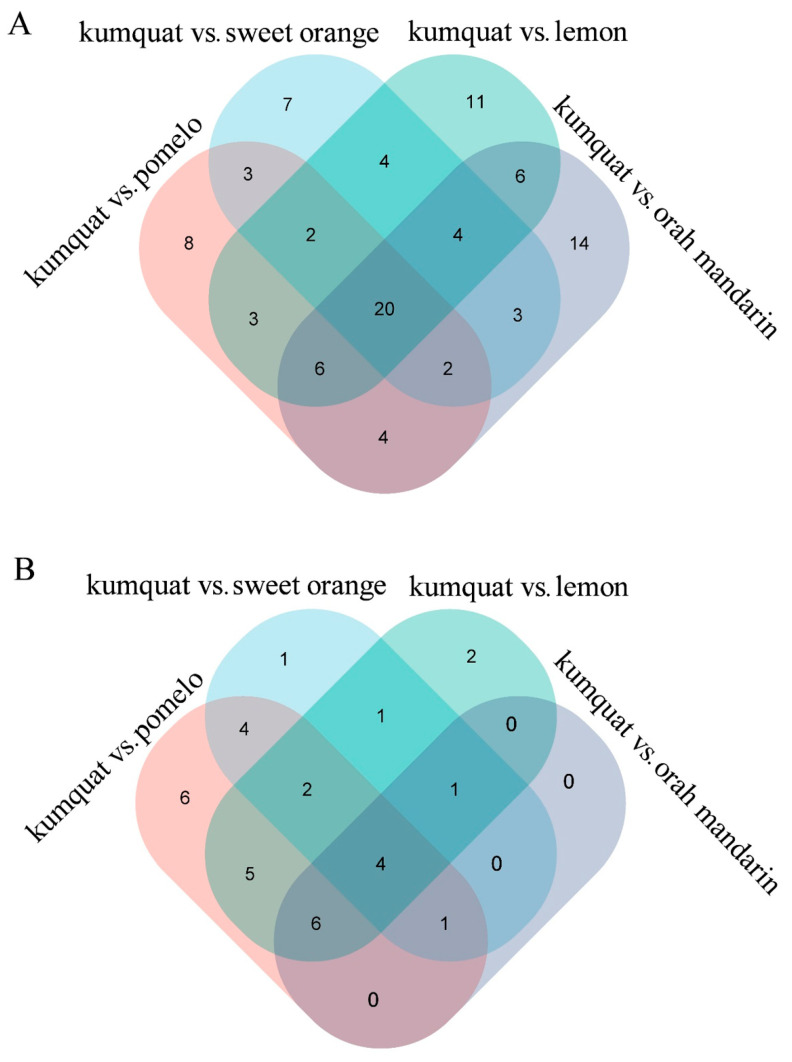
Venn diagrams of the metabolites detected in the four comparison groups. (**A**) lower levels. (**B**) higher levels. Numbers indicate the quantity of unique and shared substances.

When kumquat was used as a control, we identified 20 metabolites with lower levels and 4 metabolites with higher levels in kumquat compared with those in the other four varieties (Figure 2, Table S2). Among these metabolites with decreased concentrations, eight were lysophosphatidyl choline (LPC 18:1, 16:1 (2nd isomer), 18:1 (2nd isomer), 18:3, 16:0, 18:3 (2nd isomer), 16:1, 18:2); six were lysophosphatidyl ethanolamine (LPE 18:3 (2nd isomer), 18:3, 18:1, 18:1 (2nd isomer), 16:0, 16:0 (2nd isomer)); three were amino acids and their derivatives (L-aspartic acid, L-lysine, L-glutamine); two were nucleotides and their derivatives (cytarabine, guanosine); and one was an organic acid (citric acid). The four metabolites with higher concentrations in kumquat included two organic acids (2-hydroxyhexadecanoic acid, D-xylonic acid) and one sugar and alcohol (gluconic acid).

### 3.3. Analysis of Differential Metabolic Pathways in Different Citrus Varieties

**Figure 3:**
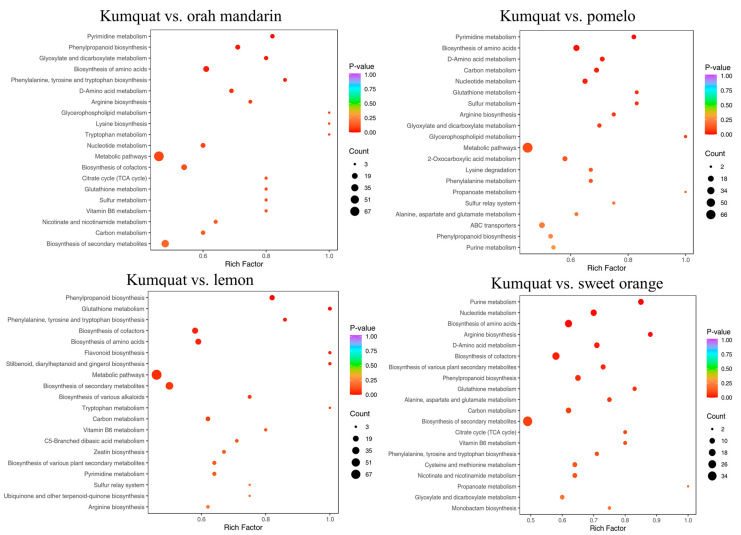
KEGG enrichment map of different citrus metabolites.

To investigate the biological implications of metabolic divergence across citrus cultivars, we conducted an enrichment analysis of differential metabolic pathways, identifying the top 20 pathways with the smallest *p*-values. Detailed KEGG pathway diagrams of the varieties are shown in Table 3, Figure 3 and Figure S4. The pairwise comparisons between the citrus varieties consistently revealed 70–79 differential metabolic pathways, with kumquat vs. pomelo and kumquat vs. sweet orange showing the highest number of 79, while lemon vs. pomelo and lemon vs. orah mandarin exhibited the fewest number of 70 (Table 3). Kumquat and other citrus varieties shared four significantly different metabolic pathways: phenylpropanoid biosynthesis, the biosynthesis of amino acids, arginine biosynthesis, and glutathione metabolism (Figure 3). Additionally, phenylpropanoid biosynthesis and the biosynthesis of amino acids were significantly different metabolic pathways that were shared by most comparisons (Figure S4).

### 3.4. Metabolic Pathway and Relative Abundance of Identified Metabolites

**Figure 4:**
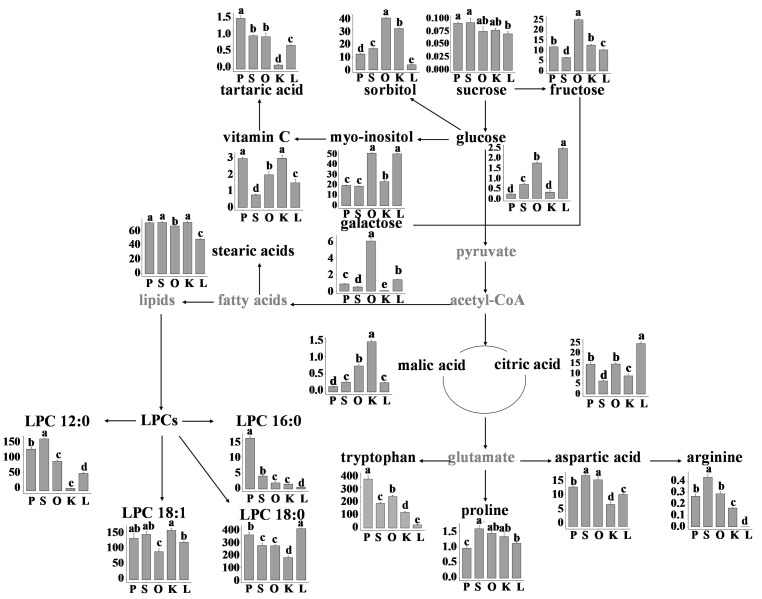
Schematic diagram of the metabolomic pathway and relative abundance of citrus flesh metabolites. P, pomelo; S, sweet orange; O, orah mandarin; K, kumquat; L, lemon. Different letters indicate significant differences, as determined using one-way ANOVA followed by Tukey’s test (*p* < 0.05).The metabolites detected in this study are set against in black.

The citrus metabolomic pathway was developed based on the metabolites detected in the citrus flesh. The relative abundances of these metabolites were then compared (Figure 4). Fructose was the predominant sugar in the citrus fruits, with orah mandarin exhibiting the greatest concentration of fructose among the five varieties. The sum of the three main sugars (galactose, fructose, and glucose) was also the highest in orah mandarin, followed by lemon, and the lowest was in sweet orange. In addition, galactose was not detected in kumquat. Two sugar alcohols, sorbitol and myo-inositol, were abundant in the citrus flesh. Like those of fructose and galactose, the amount of sugar alcohols in orah mandarin was 1.9 to 2.9 times greater than that in the other four varieties.

Significant differences were also observed in terms of amino acids and lipids, a finding that was in accordance with the results of the KEGG pathway analysis. Pomelo had the highest tryptophan content, which was 1.6 to 15.4 times greater than that of the other varieties. Sweet orange had the highest contents of aspartic acid, arginine, and proline. Additionally, arginine was not detected in lemon. LPC 18:0 was determined to be the predominant LPC in the citrus fruits, with lemon exhibiting the highest amount. Pomelo presented the greatest quantity of LPC 16:0, surpassing the other varieties by a factor of 3.93 to 29.31.

## 4. Discussion

In this study, the number of metabolites far exceeded those reported in previous studies using a combination of GC/MS, LC/MS, and HPLC [8], demonstrating the effectiveness of LC‒MS/MS. The PCA results in this study imply that there are differences in metabolites among different citrus varieties. An analysis of these differential metabolites showed that pomelo has significantly increased levels of essential amino acids (L-valine, L-methionine) and vitamin C. Both L-methionine and L-valine play positive roles in antibacterial and anti-inflammatory processes [27,28]. Additionally, orah mandarin contained significantly higher levels of the essential amino acids L-tryptophan, L-phenylalanine, and L-lysine than the other citrus varieties, providing a theoretical basis for a nutritious diet.

A previous study revealed that 27 metabolites contributed to the differences between 10 citrus varieties. These metabolites included seven sugars, five amino acids, seven lipids, and eight acidic compounds [8]. Nevertheless, our investigation identified 16 LPC species showing 3- to 29-fold variation between varieties (Figure 4), a finding absent in broader population studies. The disparity might be attributed to the distinct citrus categories used in the two studies. The identification of LPC species variation between varieties provides novel biomarkers for early-stage breeding selection. In addition, orah mandarin showed 2.9-fold higher fructose than previous reported varieties [18], suggesting cultivar-specific sugar metabolism rewiring. The difference in the fructose to citrate ratio suggests actionable strategies for metabolic engineering. The number of metabolites detected in our study was comparable with a previous report using LC‒MS/MS [10], indicating the abundance of metabolites in citrus fruits.

The analysis of the differential metabolic pathways among the different citrus varieties indicated that two pathways had significant differences in all the pairwise comparisons of the tested varieties. These pathways included phenylpropanoid biosynthesis and the biosynthesis of amino acids. Citrus species are noted by their high concentrations of phenylpropanoids, particularly flavonoids and phenolic acids, serving as a significant source of these compounds in the human diet and as a fundamental resource for the food and para-pharmaceutical industries [29]. Phenylpropanoids serve as precursors to lignins and possess structural roles, antioxidant properties, and drought resistance [30]. Phenylpropanoid biosynthesis is the primary process for the production of distinctive phenolic compounds [31], which may affect the quality of citrus pulp. Amino acids are important in neural and hormonal regulation [32,33], and the significant differences in amino acid metabolism pathways among different citrus varieties suggest that amino acids may play a key role in the development of citrus flavor and quality. Research has indicated that amino acids have significant potential in the development of functional foods [34,35]. The differences in the amino acid content also provide valuable insights into the differential utilization of amino acids in citrus.

Citric acid was the predominant organic acid found in citrus flesh, with vitamin C, malic acid, and tartaric acid being the next most abundant organic acids present. In particular, lemon had the highest citric acid content, which was approximately 1.68 to 3.86 times greater than that of the other varieties, in accordance with previous reports [36]. Kumquat and pomelo had the highest contents of vitamin C. Our previous studies also found that pomelo exhibits the highest vitamin C content, which may be linked to the expansion of the *APX* gene family [37]. Organic acids and their ratios, together with sugar contents, are primarily examined to identify the maturity and flavor criteria in citrus fruits. In general, the sugar content increases while the organic acid concentration decreases throughout fruit maturation, resulting in a sweet taste [36,38]. These metabolites may serve as targets to guide selective breeding efforts or to manipulate key metabolites by potential metabolic engineering approaches aimed at enhancing the desirable flavor profiles of citrus varieties.

## 5. Conclusions

This study systematically characterized 419 metabolites in five citrus varieties, with a prioritized analysis of primary metabolites driving flavor differentiation, while phenolic acids were screened as secondary modulators. Pomelo contained all 484 metabolites, whereas kumquat had 387. The PCA results revealed significant differences in metabolites among the different citrus varieties. An analysis of the differentially abundant metabolites revealed that the lemon vs. pomelo group presented the most differentially abundant metabolites, whereas the kumquat vs. sweet orange group presented the fewest. Pomelo fruits presented significantly increased levels of the essential amino acids L-valine, L-methionine, and vitamin C, whereas orah mandarin fruits presented significantly increased levels of L-tryptophan, L-phenylalanine, and L-lysine. Two metabolic pathways showed significant differences in all the pairwise comparisons of the tested groups. By prioritizing the primary metabolite analysis, this study deciphers the metabolic drivers of citrus flavor diversification, providing actionable insights for improving flavor profiles through targeted metabolic engineering.

## Figures and Tables

**Table 1:** List of the metabolites identified in five citrus varieties.

Class I	Class II	Pomelo	Sweet Orange	Orah Mandarin	Kumquat	Lemon	Total
Amino acids and derivatives	Amino acids and derivatives	67	67	66	63	65	67
Nucleotides and derivatives	Nucleotides and derivatives	38	38	38	37	38	38
Lipids	Free fatty acids	55	55	55	52	53	55
	Phosphatidylcholine	1	1	1	1	1	1
	Glycerol ester	15	16	16	14	15	16
	Lysophosphatidyl choline	29	29	29	29	29	29
	Lysophosphatidyl ethanolamine	28	28	28	22	24	28
Others	Saccharides and alcohols	45	45	44	40	44	45
	Vitamin	13	13	13	13	12	13
	Phenolic acids	77	76	77	70	75	77
Organic acids	Organic acids	50	50	50	46	43	50
	Total	418	418	417	387	399	419

**Table 2:** Comparison of the metabolite quantities among the five citrus varieties.

Comparisons	Higher	Lower	Sum	Comparisons	Higher	Lower	Sum
Sweet orange vs. Pomelo	30	28	58	Kumquat vs. Sweet orange	10	29	39
Orah mandarin vs. Pomelo	40	21	61	Lemon vs. Sweet orange	23	36	59
Kumquat vs. Pomelo	12	49	61	Kumquat vs. Orah mandarin	7	51	58
Lemon vs. Pomelo	28	39	67	Lemon vs. Orah mandarin	5	49	54
Orah mandarin vs. Sweet orange	42	20	62	Lemon vs. Kumquat	43	14	57

**Table 3:** Comparison of differential KEGG pathways in five citrus varieties.

	Pomelo	Sweet Orange	Orah Mandarin	Kumquat	Lemon
Pomelo	/				
Sweet orange	73	/			
Orah mandarin	78	77	/		
Kumquat	79	79	78	/	
Lemon	70	76	70	75	/

## Data Availability

The raw data of the metabolites have been released on figshare (https://figshare.com, 10 December 2024) and are available (https://doi.org/10.6084/m9.figshare.27998651, accessed on 10 December 2024).

## Supplementary Materials

The following supporting information can be downloaded at https://www.mdpi.com/article/10.3390/cimb47040223/s1.

## Abbreviations

LC‒MS/MS: liquid chromatography‒mass spectrometry; QC: quality control; PCA: principal component analysis; OPLS-DA: orthogonal partial least squares discriminant analysis; VIP: variable importance in projection; FC: fold change; KEGG: Kyoto Encyclopedia of Genes and Genomes; LPC: lysophosphatidyl choline.
