# Supplementary material for: Liquid Chromatography‒Tandem Mass Spectrometry Analysis of Primary Metabolites and Phenolic Acids Across Five Citrus Species
Source: Curr Issues Mol Biol. 2025 Mar 26;47(4):223. doi: 10.3390/cimb47040223 (PMC12026233; doi:10.3390/cimb47040223)
Supplement: Supplementary file 1 [file cimb-47-00223-s001.zip › Figure S4.pdf]

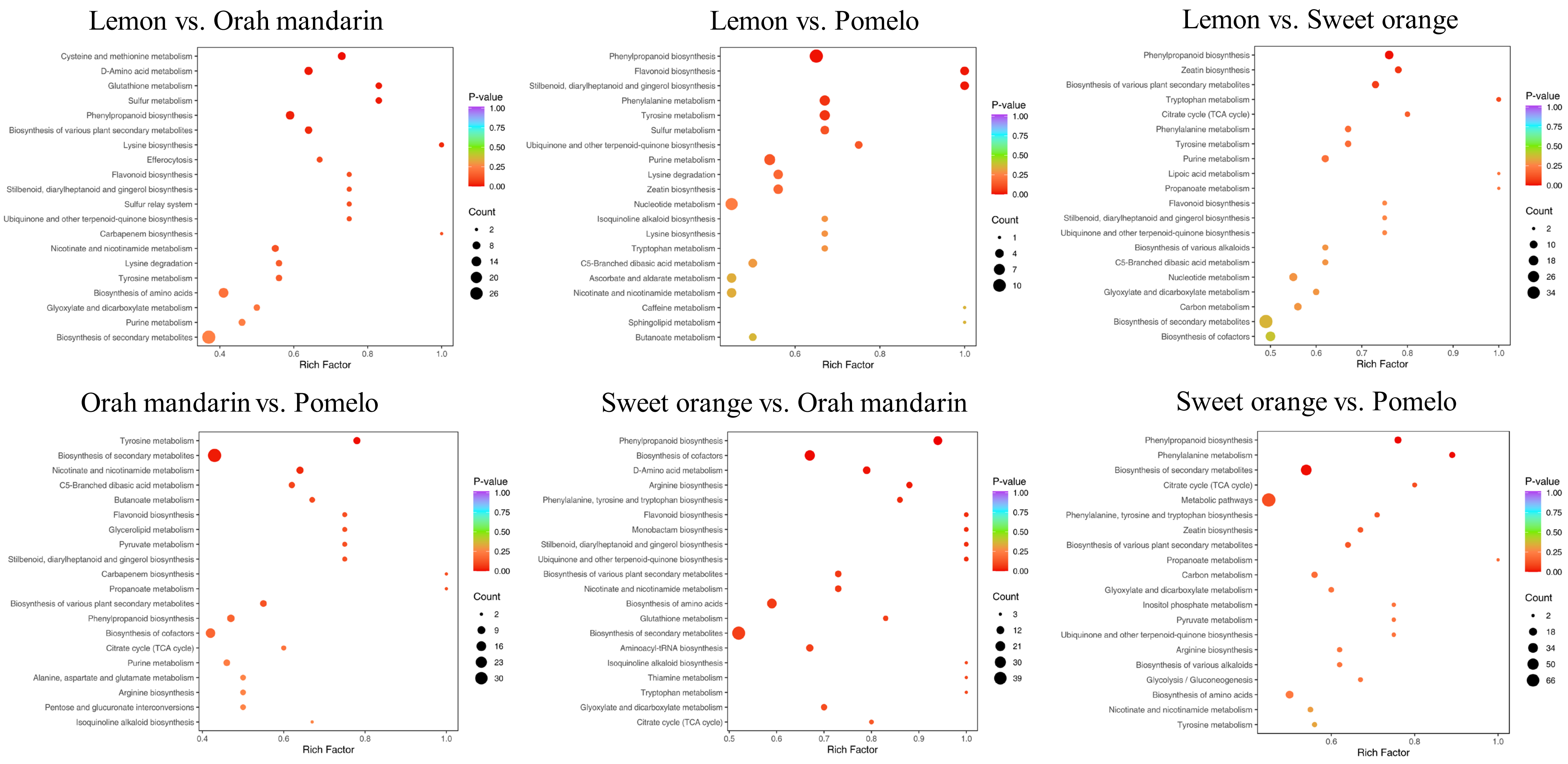

Lemon vs. Orah mandarin
Cysteine and methionine metabolism
D-Amino acid metabolism
Glutathione metabolism
Sulfur metabolism
Phenylpropanoid biosynthesis
Biosynthesis of various plant secondary metabolites
Lysine biosynthesis
Efferocytosis
Flavonoid biosynthesis
Stilbenoid, diarylheptanoid and gingerol biosynthesis
Sulfur relay system
Ubiquinone and other terpenoid-quinone biosynthesis
Carbapenem biosynthesis
Nicotinate and nicotinamide metabolism
Lysine degradation
Tyrosine metabolism
Biosynthesis of amino acids
Glyoxylate and dicarboxylate metabolism
Purine metabolism
Biosynthesis of secondary metabolites
0.4
0.6
0.8
1.0
Rich Factor
P-value
1.00
0.75
0.50
0.25
0.00
Count
2
8
14
20
26
Lemon vs. Pomelo
Phenylpropanoid biosynthesis
Flavonoid biosynthesis
Stilbenoid, diarylheptanoid and gingerol biosynthesis
Phenylalanine metabolism
Tyrosine metabolism
Sulfur metabolism
Ubiquinone and other terpenoid-quinone biosynthesis
Purine metabolism
Lysine degradation
Zeatin biosynthesis
Nucleotide metabolism
Isoquinoline alkaloid biosynthesis
Lysine biosynthesis
Tryptophan metabolism
C5-Branched dibasic acid metabolism
Ascorbate and aldarate metabolism
Nicotinate and nicotinamide metabolism
Caffeine metabolism
Sphingolipid metabolism
Butanoate metabolism
0.6
0.8
1.0
Rich Factor
P-value
1.00
0.75
0.50
0.25
0.00
Count
1
4
7
10
Lemon vs. Sweet orange
Phenylpropanoid biosynthesis
Zeatin biosynthesis
Biosynthesis of various plant secondary metabolites
Tryptophan metabolism
Citrate cycle (TCA cycle)
Phenylalanine metabolism
Tyrosine metabolism
Purine metabolism
Lipoic acid metabolism
Propanoate metabolism
Flavonoid biosynthesis
Stilbenoid, diarylheptanoid and gingerol biosynthesis
Ubiquinone and other terpenoid-quinone biosynthesis
Biosynthesis of various alkaloids
C5-Branched dibasic acid metabolism
Nucleotide metabolism
Glyoxylate and dicarboxylate metabolism
Carbon metabolism
Biosynthesis of secondary metabolites
Biosynthesis of cofactors
0.5
0.6
0.7
0.8
0.9
1.0
Rich Factor
P-value
1.00
0.75
0.50
0.25
0.00
Count
2
10
18
26
34
Orah mandarin vs. Pomelo
Tyrosine metabolism
Biosynthesis of secondary metabolites
Nicotinate and nicotinamide metabolism
C5-Branched dibasic acid metabolism
Butanoate metabolism
Flavonoid biosynthesis
Glycerolipid metabolism
Pyruvate metabolism
Stilbenoid, diarylheptanoid and gingerol biosynthesis
Carbapenem biosynthesis
Propanoate metabolism
Biosynthesis of various plant secondary metabolites
Phenylpropanoid biosynthesis
Biosynthesis of cofactors
Citrate cycle (TCA cycle)
Purine metabolism
Alanine, aspartate and glutamate metabolism
Arginine biosynthesis
Pentose and glucuronate interconversions
Isoquinoline alkaloid biosynthesis
0.4
0.6
0.8
1.0
Rich Factor
P-value
1.00
0.75
0.50
0.25
0.00
Count
2
9
16
23
30
Sweet orange vs. Orah mandarin
Phenylpropanoid biosynthesis
Biosynthesis of cofactors
D-Amino acid metabolism
Arginine biosynthesis
Phenylalanine, tyrosine and tryptophan biosynthesis
Flavonoid biosynthesis
Monobactam biosynthesis
Stilbenoid, diarylheptanoid and gingerol biosynthesis
Ubiquinone and other terpenoid-quinone biosynthesis
Biosynthesis of various plant secondary metabolites
Nicotinate and nicotinamide metabolism
Biosynthesis of amino acids
Glutathione metabolism
Biosynthesis of secondary metabolites
Aminoacyl-tRNA biosynthesis
Isoquinoline alkaloid biosynthesis
Thiamine metabolism
Tryptophan metabolism
Glyoxylate and dicarboxylate metabolism
Citrate cycle (TCA cycle)
0.5
0.6
0.7
0.8
0.9
1.0
Rich Factor
P-value
1.00
0.75
0.50
0.25
0.00
Count
3
12
21
30
39
Sweet orange vs. Pomelo
Phenylpropanoid biosynthesis
Phenylalanine metabolism
Biosynthesis of secondary metabolites
Citrate cycle (TCA cycle)
Metabolic pathways
Phenylalanine, tyrosine and tryptophan biosynthesis
Zeatin biosynthesis
Biosynthesis of various plant secondary metabolites
Propanoate metabolism
Carbon metabolism
Glyoxylate and dicarboxylate metabolism
Inositol phosphate metabolism
Pyruvate metabolism
Ubiquinone and other terpenoid-quinone biosynthesis
Arginine biosynthesis
Biosynthesis of various alkaloids
Glycolysis / Gluconeogenesis
Biosynthesis of amino acids
Nicotinate and nicotinamide metabolism
Tyrosine metabolism
0.6
0.8
1.0
Rich Factor
P-value
1.00
0.75
0.50
0.25
0.00
Count
2
18
34
50
66
