# Supplementary figures and images for: Liquid Chromatography‒Tandem Mass Spectrometry Analysis of Primary Metabolites and Phenolic Acids Across Five Citrus Species
Source: Curr Issues Mol Biol. 2025 Mar 26;47(4):223. doi: 10.3390/cimb47040223 (PMC12026233; doi:10.3390/cimb47040223)

The whole

Cross section

kumquat

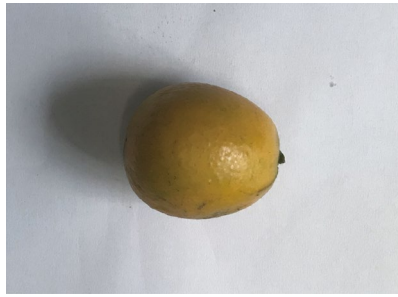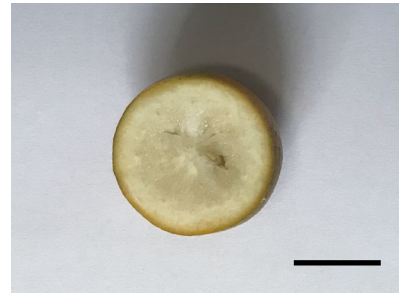

lemon

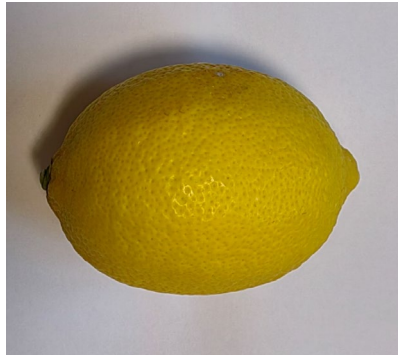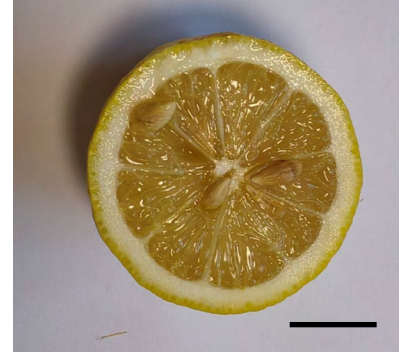

orah mandarin

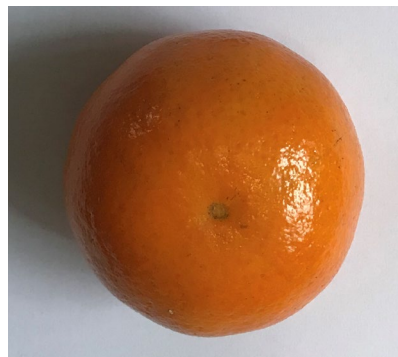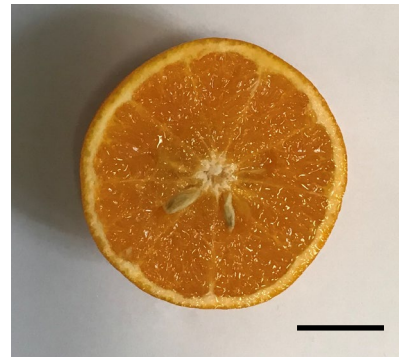

Sweet orange

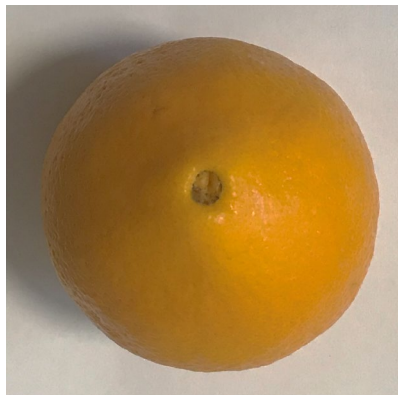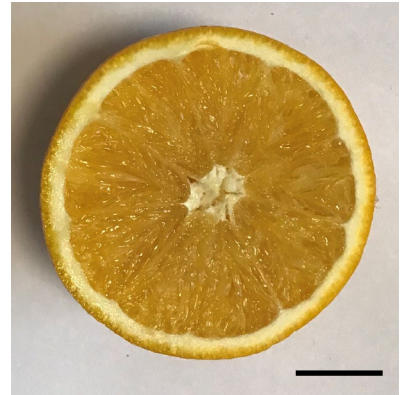

pomelo

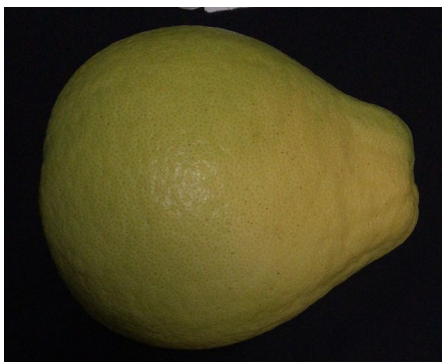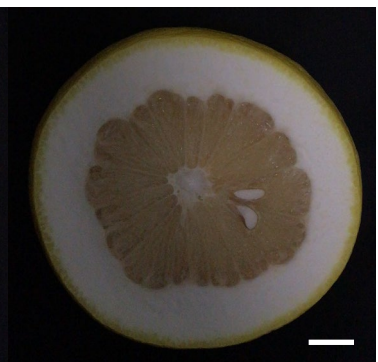

Bars = 25mm

Supplement: Supplementary file 1 [file cimb-47-00223-s001.zip › Figure S1.pdf]

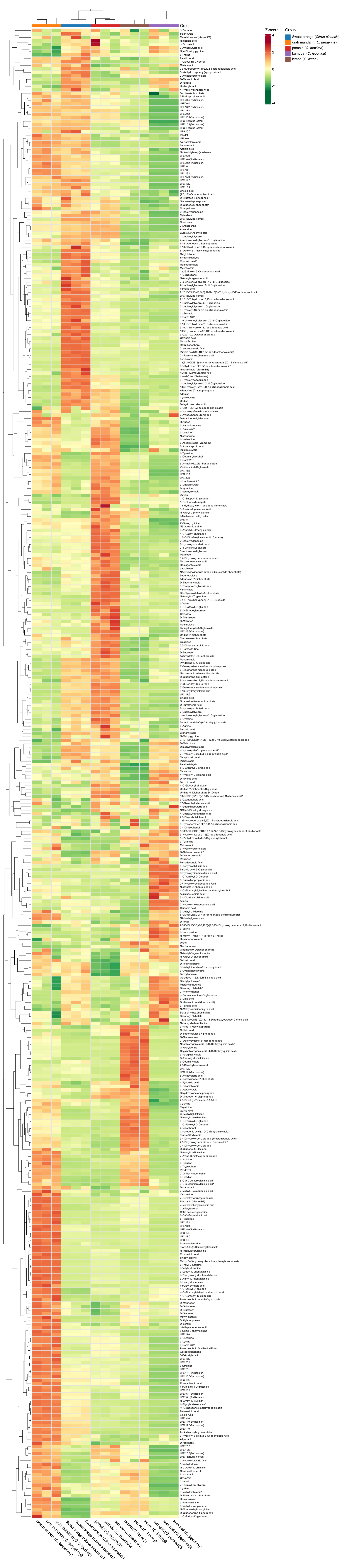

Supplement: Supplementary file 1 [file cimb-47-00223-s001.zip › Figure S2.pdf]

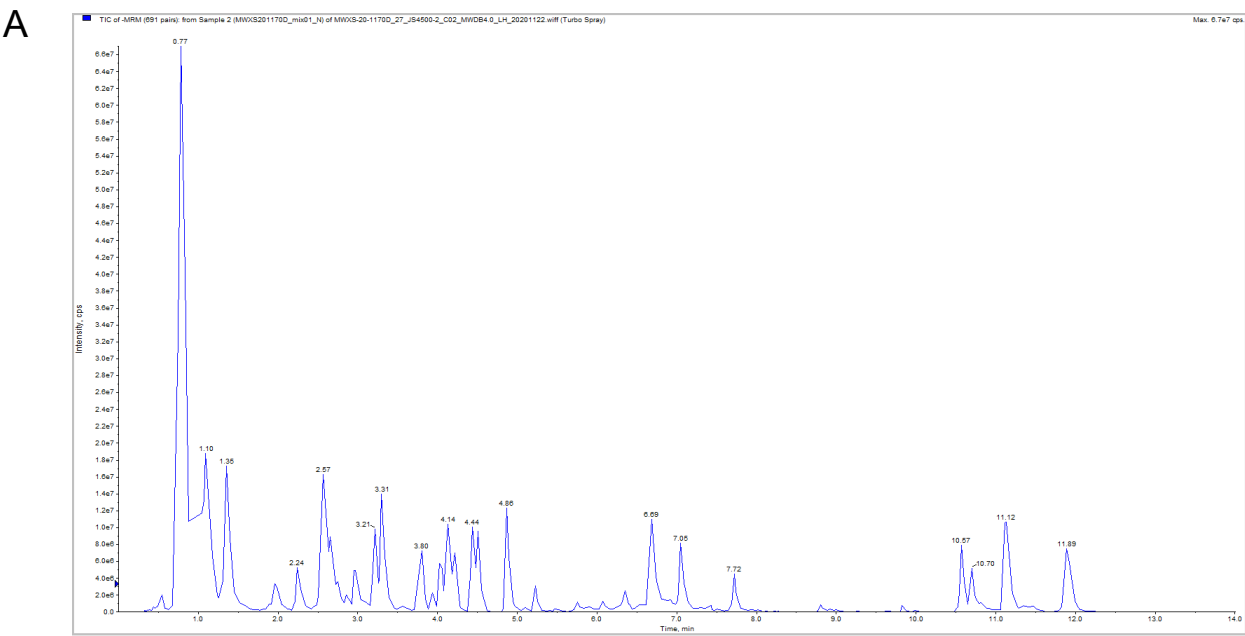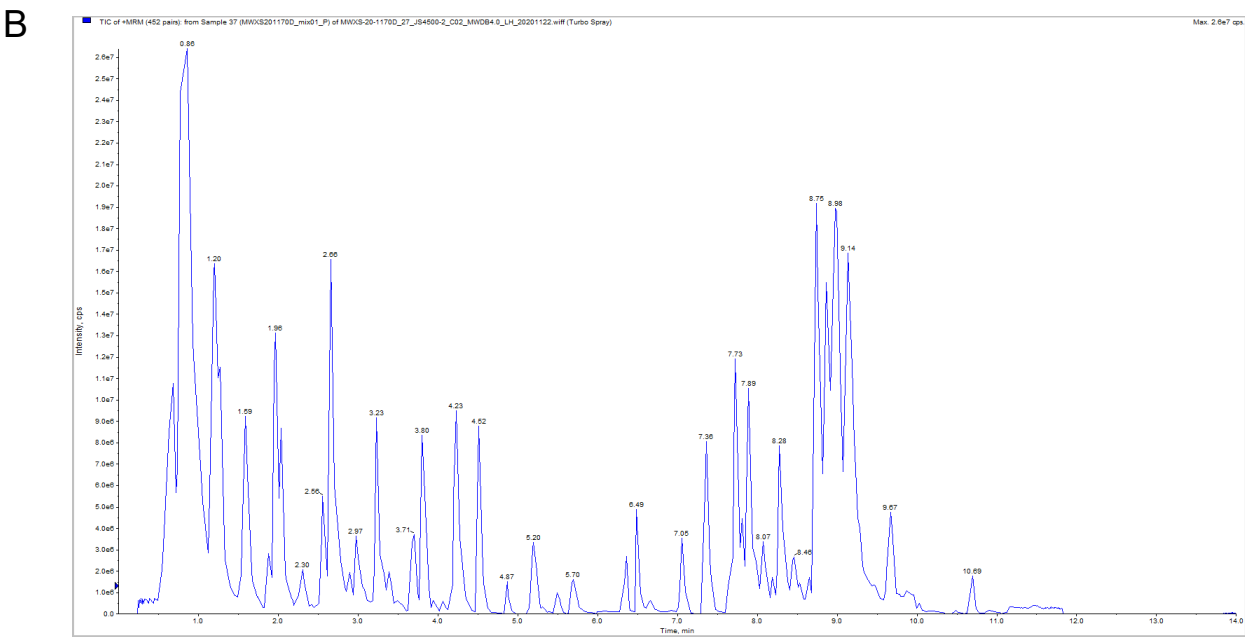

Supplement: Supplementary file 1 [file cimb-47-00223-s001.zip › Figure S3.pdf]
